# Xanthine Oxidase-Dependent Activation of NLPR3 Inflammasome in Epithelial Cells Sustains Inflammation in Inflammatory Bowel Disease

**DOI:** 10.1093/ibd/izaf231

**Published:** 2025-10-22

**Authors:** Amalia Di Petrillo, Antonella Fais, Nicola Raho, Benedetta Era, Faustina Barbara Cannea, Alessandra Padiglia, Eleonora Grecu, Daniela Murtas, Cristina Maxia, Valeria Sogos, Clara Porcedda, Pierluigi Caboni, Mauro Demurtas, Agnese Favale, Sara Onali, Massimo Claudio Fantini

**Affiliations:** Department of Medical Science and Public Health, University of Cagliari, Monserrato, Italy; Department of Life and Environmental Sciences, University of Cagliari, Monserrato, Italy; Department of Medical Science and Public Health, University of Cagliari, Monserrato, Italy; Department of Life and Environmental Sciences, University of Cagliari, Monserrato, Italy; Department of Life and Environmental Sciences, University of Cagliari, Monserrato, Italy; Department of Life and Environmental Sciences, University of Cagliari, Monserrato, Italy; Department of Biomedical Sciences, University of Cagliari, Monserrato, Italy; Department of Biomedical Sciences, University of Cagliari, Monserrato, Italy; Department of Biomedical Sciences, University of Cagliari, Monserrato, Italy; Department of Biomedical Sciences, University of Cagliari, Monserrato, Italy; Department of Biomedical Sciences, University of Cagliari, Monserrato, Italy; Department of Life and Environmental Sciences, University of Cagliari, Monserrato, Italy; Azienda Ospedaliero-Universitaria di Cagliari, Monserrato, Italy; Department of Medical Science and Public Health, University of Cagliari, Monserrato, Italy; Azienda Ospedaliero-Universitaria di Cagliari, Monserrato, Italy; Department of Medical Science and Public Health, University of Cagliari, Monserrato, Italy; Azienda Ospedaliero-Universitaria di Cagliari, Monserrato, Italy; Department of Medical Science and Public Health, University of Cagliari, Monserrato, Italy; Azienda Ospedaliero-Universitaria di Cagliari, Monserrato, Italy

**Keywords:** xanthine oxidase, inflammatory bowel disease, inflammasome

## Abstract

**Importance and Objective:**

Xanthine oxidase (XO) plays a key role in purine metabolism, catalyzing the oxidation of hypoxanthine to xanthine, and xanthine to uric acid (UA) with the production of superoxide anions. The accumulation of UA has been shown to initiate the inflammatory process through NLRP3 inflammasome, and the production of reactive oxygen species (ROS) contributes to inflammation-related tissue damage in different organs. This study aimed to investigate the role of XO in the pathogenesis of Inflammatory Bowel Disease (IBD) and the anti-inflammatory potential of XO inhibition.

**Design:**

XO expression and activity were assessed in the mucosa of moderately-to-severely active ulcerative colitis (UC) and Crohn’s disease (CD) patients. The functional role of XO, the activation of NLRP3 inflammasome, and the expression of proinflammatory cytokines were investigated in *ex vivo* intestinal organ cultures in the presence or absence of XO inhibitors.

**Results:**

*In silico* and i*n vitro* analysis showed that XO mRNA and protein expression were upregulated in the UC and CD intestinal mucosa compared to controls. XO overexpression in the inflamed mucosa was associated with increased enzymatic activity, accumulation of UA and functionally linked to NLRP3-dependent IL1beta and IL18 expression. Accordingly, XO inhibitors, Allopurinol and Febuxostat, prevented NLRP3 activation, reduced Caspase1 activity and IL1beta and IL18 expression in *ex vivo* organ cultures of inflamed intestinal mucosa from both UC and CD patients.

**Conclusions and Relevance:**

Overexpression of XO in IBD might contribute to inflammation by promoting NLRP3 inflammasome activation and proinflammatory cytokine production.

Key Messages What is already known?

- Xanthine oxidase (XO) is involved in the metabolism of purines. In IBD, pharmacologic inhibition of XO is known to enhance the immunosuppressive effect of thiopurines increasing the concentration of the active metabolite 6-thioguanine.

What is new here?

- We found that XO is overexpressed in the intestinal mucosa of IBD patients where it promotes NLRP3 inflammasome activation in intestinal epithelial cells and enhances the expression of the proinflammatory cytokines IL1beta and IL18.

How can this study help patient care?

- XO inhibition in intestinal epithelial cells may represent a novel therapeutic strategy for IBD by reducing NLRP3 inflammasome activation and proinflammatory cytokine expression.

## Introduction

Crohn’s disease (CD) and ulcerative colitis (UC), the two major forms of Inflammatory Bowel Disease (IBD), are characterized by chronically relapsing and remitting intestinal symptoms, including abdominal pain, increased stool frequency, and bloody diarrhea.^1^^,^^2^ Chronic inflammation in IBD is believed to originate from a loss of immunological tolerance towards antigens normally contained in the gut lumen, triggered by the exposure to environmental factors in genetically predisposed subjects.^3^ Indeed, these factors converge to determine a defect of the intestinal epithelial barrier and a dysregulated exposure to luminal antigens, leading to the chronic activation of the immune system, inflammation, and inflammation-related tissue damage.^4^

Xanthine oxidase (XO) together with xanthine dehydrogenase (XDH) are the two enzymatic activities of xanthine oxidoreductase (XOR), an enzyme belonging to the molybdenum hydroxylase flavoprotein family.^5^ XOR is widely expressed in human tissues, being the highest activity observed in the liver and the intestine.^6^ The oxidative form of XOR, now indicated as XO, is involved in multiple enzymatic activities, including the metabolism of purines, and its inhibition represents the mainstay therapy of gout, a disease characterized by the accumulation of uric acid (UA), the end-stage product of purine metabolism.^7^ XO has also been involved in the generation of nitric oxide and superoxide radicals during inflammation in different organs, including the gut.^8^^,^^9^ In IBD patients, the XO inhibitor Allopurinol has long been used with azathioprine to enhance its immune suppressive effect by enhancing the production of the active metabolite 6-thioguanine.^10^ However, lines of evidence suggest that the inhibition of XO activity might, per se, dampen the intestinal inflammatory process.^11^^,^^12^

Among the mechanisms involved in the proinflammatory effect of XO activity is the activation of the NLRP3 inflammasome, a protein complex responsible for the proteolytic activation of the proinflammatory cytokines IL1beta and IL8.^13–16^ NLRP3 activation occurs upon activation of damage-associated molecular patterns.^17^ However, reactive oxygen species (ROS) and UA generated by XO have been shown to contribute in different ways to NLRP3 activation and IL1beta overexpression.^13^^,^^18^

Based on these data, we hypothesized that XO could play a role in human IBD. Here, we report data demonstrating that XO expression and activity are enhanced in the inflamed mucosa of both CD and UC patients compared to healthy controls and functionally linked to UA accumulation, NLRP3 activation, and proinflammatory cytokine expression.

## Methods

### Patients

All patients with Crohn’s disease (CD) and ulcerative colitis (UC) were recruited at the Gastroenterology Department of the University of Cagliari. The diagnosis of CD and UC was based on the combination of clinical signs and symptoms, endoscopic features, and histologic results. Colonic and ileal biopsies were collected from 20 healthy subjects undergoing endoscopy for colorectal cancer screening (CTRL). Biopsies of inflamed mucosa were obtained from 20 patients with active CD and 20 patients with active UC. The endoscopic activity was calculated using the SES-CD score for CD patients and MAYO score for UC patients. Patient’s characteristics are reported in the supplemental material (Table S1).

### RNASeq Datasets Analysis

GSE11223, GSE 117993, and GSE109142 were retrieved from GEO database (http://www.ncbi.nlm.nih.gov/geo/), and Gene profile data were downloaded from GEO Profiles (https://www.ncbi.nlm.nih.gov/geoprofiles/), in which the value expression was calculated by log2 ratio (test/reference) and relative expression, as described in the original sample records (see supplemental material).

### Ex vivo Organ Culture

Fresh intestinal biopsies were cultured for 24 h in a controlled atmosphere (95% O_2_, 5% CO_2_) in organ culture medium. Tissues were incubated in the presence or absence of xanthine oxidase inhibitors (allopurinol or febuxostat) to assess inflammasome activation.

### Uric Acid Quantification

Uric acid concentrations in tissue lysates and culture supernatants were measured using ultra-high-performance liquid chromatography-tandem mass spectrometry (UHPLC-MS/MS).

### Cytokine Measurement by ELISA

Levels of IL-1β and IL-18 in supernatants from cultured biopsies were quantified using commercial enzyme-linked immunosorbent assay kits, following the manufacturer’s protocols.

### Western Blot Analysis

Total protein was extracted from mucosal biopsies and subjected to SDS-PAGE followed by immunoblotting. Protein bands for XO, NLRP3, ASC, and Caspase-1 were visualized and quantified relative to GAPDH.

### Immunohistochemistry

Paraffin-embedded tissue sections were stained for XO and inflammasome markers. Positivity was assessed semi-quantitatively based on staining intensity and distribution in epithelial and lamina propria compartments.

### Caspase-1 Activity Assay

Caspase-1 activity in tissue lysates was evaluated using a luminescence-based assay, providing relative light unit (RLU) measurements as an indicator of inflammasome activation.

### Statistical Analysis

Data was analyzed using the 2-way Student t test, Mann-Whitney U test, 1-way analysis of variance (ANOVA) test, or Spearman correlation analysis using GraphPad Prism 9. All data are presented as mean ± standard error of the mean, and a *P* value < .05 was considered statistically significant.

Detailed protocols are provided in the Supplementary Methods.

## Results

### XO Expression and Activity are Increased in the Mucosa of IBD Patients

**Figure 1.**
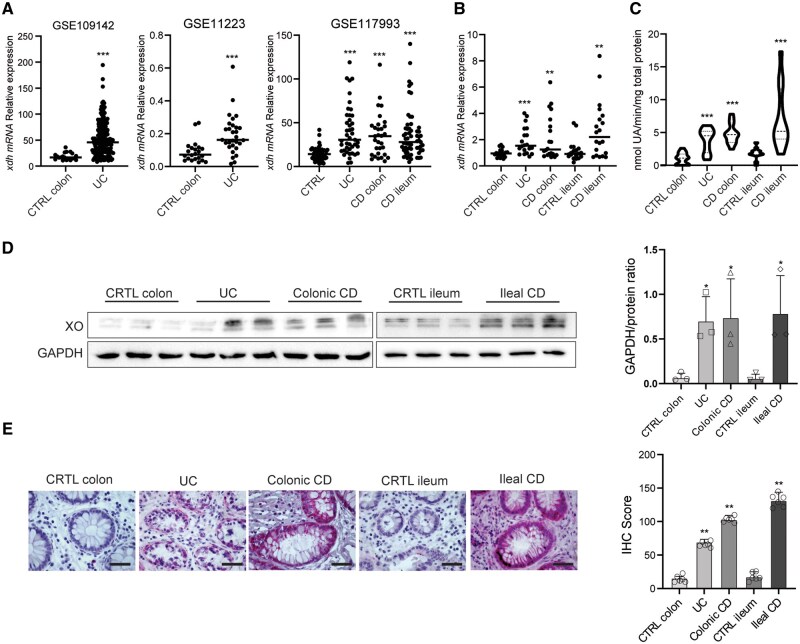
XO expression and activity in the intestinal mucosa of IBD patients. (A) Analysis of *xdh* mRNA expression in three independent GEO datasets (GSE109142 CTRL colon *n* = 20, UC = 206; GSE11223 CTRL colon *n* = 23, UC *n* = 29, and GSE117993 CTRL *n* = 55; UC *n* = 43; ileal CD *n* = 31, colonic CD *n* = 61) comparing UC, CD, and CTRL samples. (B) RT-qPCR analysis of *xdh* mRNA in colon and ileum biopsies from colonic CTRL (*n* = 20), UC (*n* = 20), colonic CD (*n* = 20), ileal CTRL (*n* = 20), and ileal CD (*n* = 20) patients. Horizontal bars indicate the median value. (C) XO enzymatic activity measured in biopsy lysates of colonic CTRL (*n* = 16), UC (*n* = 16), colonic CD (*n* = 12), ileal CTRL (*n* = 12), and ileal CD (*n* = 12), expressed as nmol UA/min/mg protein. The dashed horizontal lines in the violin plots indicate the median value and the IQR range (D) Representative Western blot showing XO protein expression levels in biopsy lysates from different groups. GAPDH expression was used as the loading control. Bar graph (right panel) shows the mean (*n* = 3) densitometric quantification of band intensity normalized to GAPDH. (E) Immunohistochemical staining of XO in paraffin-embedded mucosal biopsies from the colon (*n* = 6) and ileum (*n* = 6) of CTRL subjects and UC (*n* = 6) and ileal (*n* = 6) and colonic (*n* = 6) CD patients. Horizontal bars in the lower right corner indicate 20 µm. Quantification of staining intensity from 6 patients/group is shown in the bar graph (right panel). Bars in D and E represent mean values ± SD. (*) *P* < .05, (**) *P* < .01, (***) *P* < .001 indicate statistically significant differences vs CTRL. XO, xanthine oxidase; IBD, inflammatory bowel disease; UC, ulcerative colitis; CD, Crohn’s disease; CTRL, healthy control; UA, uric acid; SD, standard deviation.

To investigate whether XO is differentially expressed in the intestinal mucosa of patients with IBD, we first analyzed *xanthine dehydrogenase (xdh)* mRNA expression using three publicly available RNASeq datasets from Gene Expression Omnibus (GEO) database. Dataset (GSE109142) contains mRNA data from UC pediatric patients naïve to therapy and a group of non-IBD controls from a representative sub-cohort of Predicting Response to Standardized Pediatric Colitis Therapy (PROTECT).^19^ A second dataset (GSE11223) contains mRNA expression data from biopsies of adult UC patients and healthy control subjects, while GSE117993 contains data from another pediatric population of both UC and CD, and patients whose diagnosis was not confirmed were used as controls (RISK study).^20^^,^^21^ The *in silico* analysis of GSE109142 and GSE11223 showed higher relative expression of *xhd* mRNA in the colon of UC patients as compared to controls (52.23 UC vs 18.61 CTRL colon and 0.19 UC vs 0.09 CTRL colon, respectively; *P* = .0001) (Figure 1A). Similar results were obtained in the UC subgroup of patients from GSE117993 (40.24 UC vs 14.46 CTRL; *P* = .0001). In the same dataset, higher expression of *xhd* was observed in both ileal and colonic CD as compared to controls (36.17 Ileal CD vs 14.46 Ctrl *P* = .0001; 35.51 colonic CD vs 14.46 CTRL *P* = .0001).

To validate these findings in our cohort, biopsies from the inflamed areas of the colon of UC (*n* = 20) and the colon (*n* = 20) or ileum (*n* = 20) of CD patients were collected. *xhd* mRNA expression in IBD samples was compared to that of biopsies collected from the ileum (*n* = 20) and the colon (*n* = 20) of healthy patients undergoing colonoscopy for colorectal cancer screening. Results from this analysis confirmed significant higher expression of *xdh* in UC patients and colonic CD biopsies compared to colonic biopsies of control patients (1.93 UC vs 0.99 CTRL [*P* = .004] and 1.79 CD vs 0.99 [*P* = .01]), Figure 1B. Similarly, *xhd* mRNA expression was also significantly higher in the ileum of CD patients as compared to ileal controls (2.72 vs 1.26 [*P* = .004]).

XO enzymatic activity was assayed spectrophotometrically by measuring the accumulation of UA in the protein extracts of intestinal biopsies. The enzymatic activity of XO was significantly higher in biopsies of UC and colonic CD compared to healthy colonic controls, (3.86 nmol UA/min/mg in UC, 4.82 in CD vs 1.03 in CTRL, *P* < .001) and in CD ileum as compared to ileal controls (8.41 vs 1.83 in CTRL, *P* < .001), Figure 1C. Finally, higher XO expression was confirmed at protein level in the mucosa of UC and both ileal and colonic CD as compared to CTRL by Western blotting (Figure 1D). Also, IHC of sections obtained from intestinal biopsies confirmed higher expression of XO in IBD as compared to controls (Figure 1E) and the staining was prevalently localized in the epithelial cell cytoplasm (Figure S1).

These data demonstrate higher XO expression and activity in the intestinal epithelial cells of IBD patients as compared to uninflamed controls, thus suggesting its possible role in the pathogenesis of the inflammatory process.

### NLRP3 Inflammasome is Hyperactivated in XO-Expressing Epithelial Cells of IBD Patients

**Figure 2.**
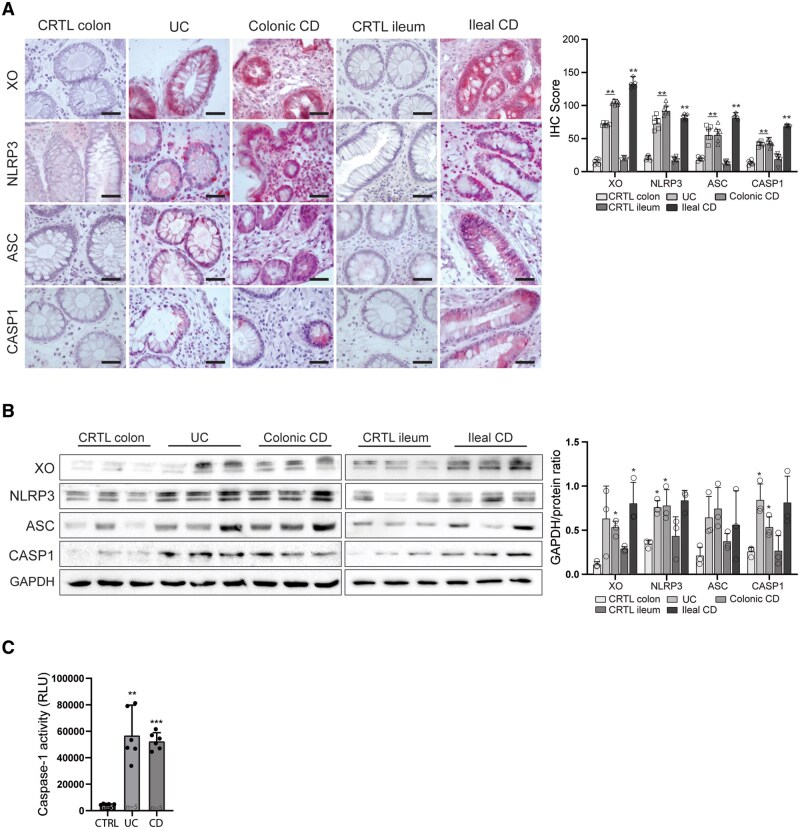
NLRP3 inflammasome components and caspase-1 activity in the intestinal mucosa of IBD patients. (A) Representative immunohistochemical staining of NLRP3, ASC, and Caspase-1 in biopsies from the colon (*n* = 6) and ileum (*n* = 6) of CTRL subjects and UC (*n* = 6), and ileal (*n* = 6) and colonic (*n* = 6) CD patients. Horizontal bars in the lower right corner indicate 20 µm. Bar graphs (right panel) display semi-quantitative scoring of staining intensity from 6 patients/group. (B) Representative Western blot of NLRP3, ASC, and caspase-1 protein expression in mucosal biopsy lysates. GAPDH was used as a loading control. Bars graphs show the mean (*n* = 3) densitometric quantification of band intensity normalized to GAPDH (right panel). (C) Caspase-1 enzymatic activity measured in total tissue lysates using a luminescence-based assay. Data are presented as relative luminescence units (RLU) evaluated in CTRL (*n* = 6), subjects and in UC (*n* = 6) and CD (*n* = 6) patients. Data are presented as mean ± SD. Statistical significance was determined by appropriate tests as indicated. (*) *P* < .05, (**) *P* < .01, (***) *P* < .001 indicate statistically significant differences vs CTRL. NLRP3, NOD-like receptor family pyrin domain containing 3; ASC, apoptosis-associated speck-like protein containing a CARD; SD, standard deviation; CTRL, healthy control; UC, ulcerative colitis; CD, Crohn’s disease; RLU, relative luminescence units

The NLRP3 inflammasome is an intracellular proteolytic complex formed by different subunits, including NLRP3 and ASC, responsible for the cleavage of pro-Caspase 1 into its active form, which is responsible for the proteolytic activation of IL1beta and IL18, two proinflammatory cytokines upregulated in IBD. Since XO-derived UA and ROS have been shown to induce NLRP3 inflammasome activation,^13^^,^^18^ we investigated XO expression and inflammasome activation in mucosal biopsies of UC and CD patients. Protein expression pattern of NLRP3, ASC and activated Caspase 1 mirrored that of XO being higher in IBD mucosal biopsies as compared to controls as shown by IHC (Figure 2A). Similarly to XO, also NLRP3, ASC and Caspase 1 had a prevalent localization in the cytoplasm of epithelial cells though scattered positive cells were also detected in the lamina propria. The expression of the inflammasome subunits was further analyzed by Western blotting, confirming higher expression of NLRP3, ASC, and Caspase 1 in IBD as compared to controls (Figure 2B). To confirm that higher expression of NLRP3 inflammasome components corresponded to higher Caspase 1 activation, we measured Caspase 1 activity in biopsies of IBD patients and controls. We found that Caspase-1 activity was significantly increased in patients with UC and CD compared to control patients (5652.0 ± 1558.3 RLU UC, and 5213.0 ± 519.4 RLU CD vs 405.0 ± 40.2 RLU CTRL; *P* < .01 and *P* < .001 for UC and CD vs CTRL, respectively; Figure 2C).

The elevated Caspase-1 activity further supports the hypothesis that the NLRP3 inflammasome hyperactivation in the intestinal mucosa of IBD patients might be sustained by XO hyperexpression and activity in the same cell compartment.

### Inhibition of XO Reduces Inflammasome Activation in Ex Vivo Organ Cultures from IBD Patients

**Figure 3.**
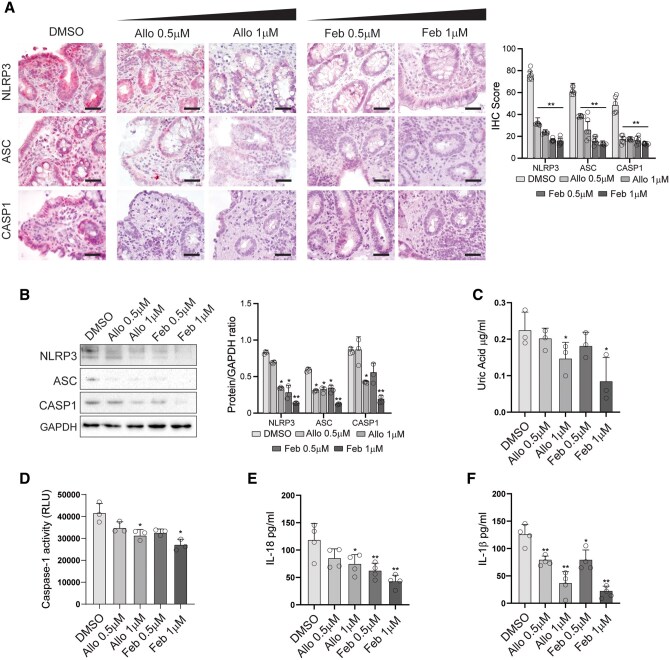
XO inhibition reduces inflammasome activation and cytokine production in CD mucosal explants. (A) NLRP3, ASC, and caspase-1 immunohistochemical staining of sections obtained from organ cultures of CD colonic biopsies stimulated with 0.5 or 1 µM allopurinol or febuxostat, or left unstimulated as indicated. DMSO-treated biopsies served as vehicle controls. Bar graphs (right panel) show semi-quantitative scoring of staining intensity of duplicate experiments from CD (*n* = 3) patients. (B) Western blot analysis of NLRP3, ASC, and caspase-1 protein expression in treated biopsy lysates. GAPDH was used as the loading control. Bar graphs (right panel) show densitometric quantification of bands normalized to GAPDH from three independent experiments. (C) Uric acid concentration in culture supernatants measured after treatment with XO inhibitors. (D, E) Concentrations of IL -1β and IL -18 in supernatants assessed by ELISA following allopurinol or febuxostat treatment. (F) Caspase-1 enzymatic activity measured in total tissue lysates using a luminescence-based assay. Data are presented as relative luminescence units (RLU). Data are presented as mean ± SD of independent experiments (*n* = 3 in C; *n* = 4 in D and E; *n* = 3 in F). (*) *P* < .05, (**) *P* < .01, (***) *P* < .001 indicate statistically significant differences vs CTRL. CD, Crohn’s disease; XO, xanthine oxidase; IL, interleukin; ELISA, enzyme-linked immunosorbent assay; GAPDH, glyceraldehyde-3-phosphate dehydrogenase; RLU, relative luminescence units; SD, standard deviation; DMSO, dimethyl sulfoxide.

To functionally link XO activity to NLRP3 inflammasome activation, we assessed whether inhibition of XO could reduce inflammasome activation and expression of IL1beta and IL18. To this end, we first treated organ cultures of mucosal biopsies from CD patients with allopurinol or febuxostat, two inhibitors of XO activity. As expected from the block of XO activity, UA concentration was progressively reduced by higher concentrations of both allopurinol and febuxostat (Figure 3C). Immunohistochemical analysis (Figure 3A) showed that NLRP3, ASC, and Caspase 1 protein expression was suppressed in organ cultures from IBD patients treated with either allopurinol or febuxostat in a dose-dependent manner as compared to those left untreated (DMSO vehicle only). Western blot analysis confirmed a significant reduction in NLRP3, ASC, and Caspase 1 protein expression following treatment with XO inhibitors (Figure 3B). Densitometric analysis of protein levels normalized to GAPDH demonstrated a dose-dependent suppression, with the highest inhibition observed at 1 µM concentrations of both allopurinol and febuxostat.

In agreement with the inhibition of NLPR3 inflammasome expression, inhibition of XO by either allopurinol or febuxostat significantly reduced Caspase 1 activity (Figure 3D) and the expression of active IL1beta and IL18 in a dose-dependent manner (Figure 3E and F).

Similar results were obtained in organ cultures of UC biopsies, which are reported in Figure S2.

Despite the functional link between XO activity and Caspase1 activation, a direct correlation between XO activity and IL18 used as surrogate marker of Caspase1 activity was not observed (Figure S3).

**Figure 4.**
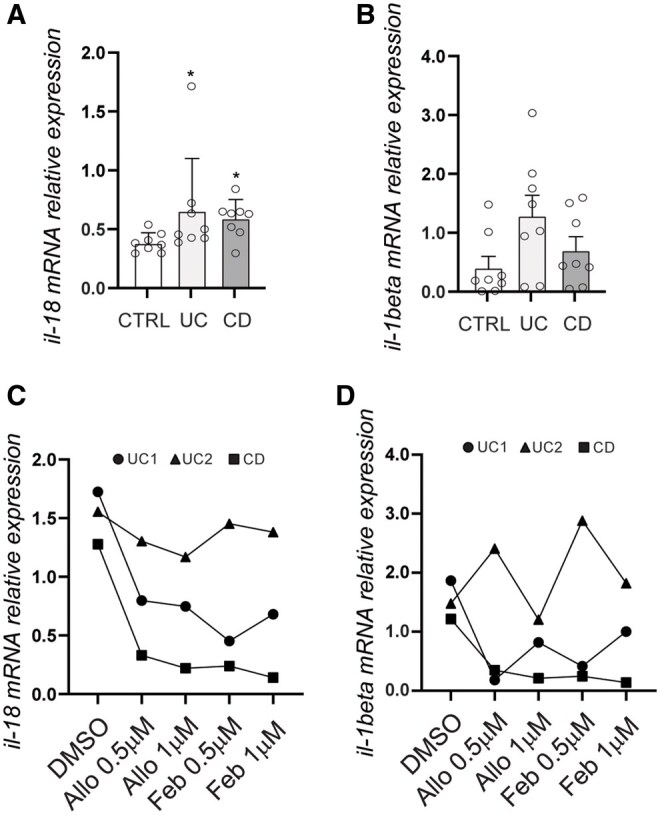
XO inhibition downregulates IL18 and IL1beta mRNA expression in inflamed mucosal explant from IBD patients. *Il18* (A) and *il1beta* (B) *mRNA* relative expression in mucosal biopsies from UC (*n* = 8), CD (*n* = 7) and control (CTRL; *n* = 7) subjects.*il18* (C) and *il1beta* (D) relative mRNA expression in organ cultures from 2 UC and 1 CD patients treated with allopurinol or febuxostat (0.5 or 1 µM as indicated) or left unstimulated (DMSO only). (*) *P* < .05. CD, Crohn’s disease; UC ulcerative colitis, CTRL, healthy control; DMSO, dimethyl sulfoxide.

Since XO activity and ROS production have been shown to activate intracellular signaling pathways including NFkB, a transcription factor involved in the expression of several proinflammatory cytokines,^11^^,^^12^ we investigated whether XO activity was also involved in the upregulation of pro-18 and pro-IL1beta. To this end we first measured the expression of IL18 and IL1beta mRNA, which encode for pro-18 and pro-IL1beta, in mucosal biopsies from IBD and control subjects. IL18 mRNA was significantly upregulated in both CD and UC patients as compared to controls (Figure 4A). The same pattern of expression was also observed for IL1beta (Figure 4B). The up regulation of both IL18 and IL1beta was, at least in part, dependent on XO activity as demonstrated by their downregulation after stimulation of mucosal organ cultures of UC and CD patients with different doses of allopurinol or febuxostat (Figure 4C and D).

These data demonstrate that XO promotes the expression of the proinflammatory cytokines IL1beta and IL18 in the mucosa of IBD patients by acting at transcriptional, and post-transcriptional level via NLRP3 inflammasome activation.

## Discussion

In this study, we showed that XO is overexpressed in the inflamed mucosa of IBD patients, contributing to inflammation, at least in part, by NLRP3 inflammasome activation and expression of the proinflammatory cytokines IL1beta and IL18. XOR is a homodimer of approximately 300 kDa composed of two domains. The largest domain harbors both the xanthine dehydrogenase and xanthine oxidase activity. The balance between these two enzymatic activities is determined by reversible or irreversible post-transcriptional modifications occurring at the level of two critical cysteine residues. When the oxidation of the sulfhydryl groups of these cysteine residues occurs, such as during inflammation, the enzymatic activity is reversibly shifted towards oxidation. Alternatively, the oxidative shift can become irreversible upon proteolytic cleavage of XOR at the site of cysteine residues.^7^^,^^22^

High oxidative activity of XOR has been involved in the pathogenesis of different inflammatory conditions, including ischemia-reperfusion injury, atherogenesis and arterial thrombosis, uremic kidney injury, COVID-19, and cancer.^23–27^ Hypoxia and proinflammatory cytokines are two known inducers of XO in different tissues, where it is responsible for the accumulation of UA and production of high concentrations of reactive oxygen (ROS) and nitrogen (RNS) species.^28^^,^^29^ High concentrations of ROS and RNS can induce damage to several intracellular structures by peroxidation, causing cell death and DNA damage.^30^ Moreover, high XO activity has been shown to indirectly induce inflammation through NLRP3 inflammasome activation and HIF1-alpha stabilization.^13–15^^,^^18^^,^^31^

In our cohort of mildly to severely active IBD patients, we demonstrated high expression of XOR at the mRNA and protein level and higher oxidative activity in both UC and CD as compared to healthy subjects. Our expression data were consistent with those generated by the *in silico* analysis of three independent RNASeq datasets, confirming higher *xdh* expression in both CD and UC as compared to noninflamed controls. Comparative XO expression analysis between IBD and healthy subjects was previously reported. Kruidenie et al analyzed XO expression in surgical specimens from CD, UC, and macroscopically normal colon areas of patients undergoing colorectal cancer surgery. In this study, XO expression was unaffected by inflammation in both UC and CD and comparable to that of control subjects, although they tended to be higher in CD.^32^ However, the analysis was based exclusively on IHC, and neither XO mRNA expression nor oxidative enzymatic activity was evaluated in this study. Reynolds et al failed to demonstrate increased oxidative activity in a small group of mildly to moderately active UC patients.^33^ The small number of patients enrolled in this study and the known variability of XO activity in IBD patients might explain this odd result. Indeed, in Reynold’s paper^34^ and in contrast with our cohort, only patients with mild to moderate endoscopic disease activity were enrolled. Since a correlation between XOR expression and the grade of endoscopic disease activity in our cohort of UC patients was observed (data not shown), the missing difference could be related to the absence of severe inflammation in the biopsies analyzed in this study. Moreover, we observed higher protein expression and oxidative activity in IBD patients, thus supporting that XO activity is enhanced in the inflamed mucosa of both CD and UC patients.

XO is physiologically expressed in epithelial cells in normal conditions where it exerts an antimicrobial activity mediated by ROS production (reviewed in Ref. 35). However, the enhanced overexpression of XO observed in IBD patients fits well with the mucosal environment of active IBD patients characterized by hypoxia and accumulation of proinflammatory cytokines. Indeed, the state of hypoxia of the inflamed mucosa, driven by the high consumption of oxygen by infiltrating neutrophils and macrophages, may well contribute to increasing XO expression.^36^ Moreover, proinflammatory cytokines such as TNF-alpha, IL1beta, and IFN gamma, highly expressed in IBD, have been shown to induce the transcription of *xhd* gene and enhance XO protein synthesis in epithelial cells.^28^ Therefore, the high expression of XO and oxidative activity observed in biopsies of IBD patients might represent the biological consequence of the hypoxic inflammatory environment characterizing both UC and CD patients. Accordingly, XO was upregulated in different experimental models of colitis where its functional role was investigated. In rodent models of colitis characterized by epithelial barrier damage, XO expression and activity were increased.^11^^,^^12^^,^^30^ Interestingly, when treated with XO inhibitors, DSS-treated mice had less expression of the transcription factor NFkB in the epithelial cells and of the proinflammatory cytokines IL1beta, IL6, and TNF-alpha. Moreover, hypoxia-induced XOR accumulation in the nucleus of epithelial cells caused DNA oxidative damage and increased HIF1-alpha protein levels. Administration of Allopurinol or Febuxostat ameliorated colitis severity, rebalancing the oxidative environment generated during inflammation. Increased XO activity and UA accumulation in the gut have also been associated with the alteration of gut barrier permeability induced by *Saccharomyces cerevisiae*, a yeast frequently observed in the gut of patients affected by CD.^37^ XO inhibition prevented *Saccharomyces cerevisiae*-induced gut permeability and ameliorated colitis severity in mice treated with DSS. Finally, XO inhibitors reduced DNA damage and prevented colon carcinogenesis in a mouse model of colitis-associated colorectal cancer.^38^

NLRP3 inflammasome is responsible for the proteolytic activation of IL1beta and IL18, and it has been shown to play a role in the pathogenesis of IBD.^39^ While macrophages mainly express IL1beta, and it has been shown to promote the expression of other key proinflammatory cytokines such as TNF-alpha and IL6, IL18 is expressed by intestinal epithelial cells where it is involved in cell proliferation and wound healing. NLRP3 is also involved in the induction of pyroptosis, a programmed cell death modality playing a key role in the pathogenesis of IBD.^40^ NLRP3 inflammasome activation is induced by the exposure to several environmental factors, including pathogen- and danger-associated molecular patterns (PAMPs and DAMPs). These different extracellular stimuli can activate NLRP3 via three main intracellular pathways: increase of K+ influx, lysosome damage, and ROS production. Accordingly, the reduced NLRP3 and NLRP3-dependent cytokines expression observed in our experiments might be dependent on reduced intracellular ROS production secondary to XO inhibition. Moreover, the reduced intracellular accumulation of UA after XO inhibition could remove another known NLRP3 activation trigger.^13^^,^^15^ Nevertheless, the observed absence of correlation between XO and Caspase1 activity might be due to the co-presence of other XO-independent NLRP3-activating stimuli during the inflammatory process.

Finally, our data indicate that XO activity also promotes the expression of IL18 and IL1beta mRNA increasing the pool of pro-IL18 and pro-IL1beta substrates available for NLRP3-dependent Caspase1 cleaving activity further supporting the proinflammatory role of XO via the expression of IL18 and IL1beta.

To the best of our knowledge, we provide for the first time evidence of an immunomodulatory effect of XOR inhibition in human IBD. Allopurinol is currently given alongside thiopurines (azathioprine and 6-mercaptopurine) in IBD patients to increase the bioavailability of the active metabolite 6-thioguanine. However, the therapeutic effect of allopurinol alone in IBD has never been assessed.^41^^,^^42^ Our data support the hypothesis that XOR inhibition might help control intestinal inflammation by reducing oxidative stress and modulating relevant inflammatory mechanisms such as NLRP3 inflammasome activation in intestinal epithelial cells, thus providing a rationale to repurpose XO inhibition as a novel mechanism to dampen intestinal inflammation in IBD.

## Supplementary Material

izaf231_Supplementary_Data
