# Supplementary material for: Xanthine Oxidase-Dependent Activation of NLPR3 Inflammasome in Epithelial Cells Sustains Inflammation in Inflammatory Bowel Disease
Source: Inflamm Bowel Dis. 2025 Oct 22;31(12):3398–406. doi: 10.1093/ibd/izaf231 (PMC12688067; doi:10.1093/ibd/izaf231)
Supplement: izaf231_Supplementary_Data [file izaf231_supplementary_data.zip › Supplementary Legends clean copy.docx]

**Supplementary Figure 1.** Immunohistochemical localization of XO in intestinal biopsies from IBD patients.

Representative images of XO immunohistochemical staining of colonic and ileal biopsies from CTR, UC, and CD patients. Images were acquired at original magnification of X63 in oil immersion. Arrows and dashed lines highlight positive cells. Layout: upper left (**a**), colonic CTRL; upper right (**b**), UC; lower left (**c**), colonic CD; lower right (**d**), ileal CD. XO, xanthine oxidase; UC, ulcerative colitis; CD, Crohn’s disease; CTRL, healthy control.

**Supplementary Figure 2.** XO inhibition reduces inflammasome activation and cytokine production in UC mucosal explants.

**(A)** NLRP3, ASC, and caspase-1 immunohistochemical staining of sections obtained from organ cultures of UC colonic biopsies stimulated with 0.5 or 1 µM of allopurinol or febuxostat, or left unstimulated as indicated. DMSO-treated biopsies served as vehicle controls. Bar graphs (right panel) show semi-quantitative scoring of staining intensity from UC (n=3) patients. **(B)** Western blot analysis of NLRP3, ASC, and caspase-1 protein expression in treated biopsy lysates. GAPDH was used as the loading control. Bar graphs(right panel) show densitometric quantification of bands normalized to GAPDH from 3 independent experiments. **(C)** Uric acid concentration in culture supernatants measured after treatment with XO inhibitors. **(D, E)** Concentrations of IL-1β and IL-18 in supernatants assessed by ELISA following allopurinol or febuxostat treatment. **(F)** Caspase-1 enzymatic activity measured in total tissue lysates using a luminescence-based assay. Data are presented as relative luminescence units (RLU). Data are presented as mean ± SD (n=3 in C; n=4 in D and E; n=3 in F). (*) p < 0.05, (**) p < 0.01, (***) p < 0.001 indicate statistically significant differences *versus* CTRL. UV, ulcerative colitis; XO, xanthine oxidase; IL, interleukin; ELISA, enzyme-linked immunosorbent assay; GAPDH, glyceraldehyde-3-phosphate dehydrogenase; RLU, relative luminescence units; SD, standard deviation; DMSO, dimethyl sulfoxide.

**Supplementary Figure 3.** Correlation between XO activity and IL18 expression.

Correlation analysis between XO activity expressed as nmol UA/mg total protein) and IL18 concentration (pg/ml) in pooled biopsies from CTRL (n=4), CD (n=5) and UC (n=4) subjects. XO, xanthine oxidase; UC, ulcerative colitis; CD, Crohn’s disease; CTRL, healthy control.
