# Supplementary material for: Xanthine Oxidase-Dependent Activation of NLPR3 Inflammasome in Epithelial Cells Sustains Inflammation in Inflammatory Bowel Disease
Source: Inflamm Bowel Dis. 2025 Oct 22;31(12):3398–406. doi: 10.1093/ibd/izaf231 (PMC12688067; doi:10.1093/ibd/izaf231)
Supplement: izaf231_Supplementary_Data [file izaf231_supplementary_data.zip › Suppl. Table 1. Clinical_Demographic_Characteristics.docx]

# Suppl. Table1. Clinical and Demographic Characteristics.

|  | **HC (n=20)** | **CD (n=20)** | **UC (n=20)** |
| --- | --- | --- | --- |
| **Age** (Mean ± SD) | 37±9 | 51.3±20.2 | 42.9±12.7 |
| **Male** (%) | 59 | 43 | 53 |
| **Disease Duration** (Mean ± SD, years) | - | 11±3 | 7±4 |
| **PCR** (Median [IQR]) | - | 8.8 [0.3-33] | 6.1 [0.8-25] |
| **Calprotectin** (Median [IQR]) | - | 95.0 [64.0 - 97.5] | 280.5 [60.7-821.3] |
| **Ongoing Therapy** (%) |  |  |  |
| Steroids | - | 12 | 30 |
| Advanced Therapy | - | 30 | 13 |
| Immunosuppressants | - | 5 | 2 |
| 5-ASA | - | 15 | 20 |
| **Disease Location** (%) |  |  |  |
| L1 Ileal | - | 10 | - |
| L2 Colonic | - | 50 | - |
| L3 Ileo-colonic | - | 40 | - |
| L4 Proximal loc. | - | 0 | - |
| **Disease Extent** (%) |  |  |  |
| E1 proctitis | - | - | 38 |
| E2 Left-sided | - | - | 37 |
| E3 Extensive | - | - | 21 |
| **HBI** (Mean ± SD) | - | 1.17 ±0.75 | - |
| **Mayo score** (Mean ± SD) | - | - | 1.93±2.26 |
| **Endoscopic Activity** (%) | N/A | SES-CD >7: 40 | MES 2-3: 68 |

*CD, Crohn’s disease; UC, ulcerative colitis; HBI, Harvwey-Bradshaw Index; 5-ASA, 5-Aminosalicylic Acid; SES-CD, Simple Endoscopic Score for Crohn’s Disease; MES, Mayo endoscopic score.*
