# Supplementary material for: Xanthine Oxidase-Dependent Activation of NLPR3 Inflammasome in Epithelial Cells Sustains Inflammation in Inflammatory Bowel Disease
Source: Inflamm Bowel Dis. 2025 Oct 22;31(12):3398–406. doi: 10.1093/ibd/izaf231 (PMC12688067; doi:10.1093/ibd/izaf231)
Supplement: izaf231_Supplementary_Data [file izaf231_supplementary_data.zip › Supplemental Material and Methods.docx]

**Supplemental material and methods:**

*Patients: inclusion and exclusion criteria*

All patients and control subjects were included after having obtained written informed consent. They have to be older than 18 years, confirmed diagnosis of UC and CD according to international guidelines.

Patients with gastrointestinal pathologies different from CD and UC and taking drugs that can influence in vitro studies, such as XO inhibitors (allopurinol or febuxostat) were excluded

*RT-qPCR analysis*

Total RNA was extracted by Trizol® (Thermo Fisher Scientific, Waltham, MA, USA), according to the manufacturer’s protocol. The concentration and purity of isolated RNA were estimated using the ND-1000 microspectrophotometer (Thermo). The RNA purity was evaluated using the ratio of A260/A280 (1.8–2.0). Two μg of RNA was utilized for cDNA synthesis using RevertAid RT Reverse Transcription Kit (Thermo). Aliquots of PCR products were visualized by electrophoresis on 2% agarose gels. qPCR was performed as follows: initial denaturation at 95 ◦C for 10 min; 40 cycles of denaturation at 95 ◦C for 15 s; and an annealing extension at 60 ◦C for 30 s. The absorbance values were measured at the extension stage. Data were presented in arbitrary units and calculated by 2−ΔΔCT method. Primers used in this study are listed in supplementary material and methods.

*Protein extraction and Quantification*

Biopsies were processed using two different extraction buffers depending on the downstream application. For enzymatic activity assays, tissues were homogenized in 50 mM phosphate buffer (pH 7.4) with 1 mM EDTA and 0.1% Triton X-100, ensuring enzyme stability. For Western blot analysis, proteins were extracted using RIPA buffer (Thermo Fisher Scientific) supplemented with protease and phosphatase inhibitors (Roche). Homogenization was performed at 4°C, followed by centrifugation at 14,000 × g for 15 minutes. The supernatant was collected and stored at -80°C until use.

Protein concentration was measured using the Bradford Protein Assay (Bio-Rad), with absorbance read at 595 nm using a spectrophotometer. A BSA standard curve was used for quantification, and protein concentrations were normalized before analysis.

*Xanthine Oxidase Activity quantification.*

XO activity of HC and IBD patients will be detected spectrophotometrically. This method is based on the capacity of XO to convert xanthine into UA. The reaction mix contained 0,1mg/ml of biopsy protein lysate and 150μM of xanthine. The activity of the XO was determined spectrophotometrically (Varian Cary 50) by measuring the production of UA at 295 nm, for 30 minutes. XO activity was expressed as nmol of UA/min/mg of total protein.

*Ex-vivo organ culture*

Intestinal biopsies from patients were cultured for 24 hours in a 24-well plate with 250 µL RPMI 1640 GlutaMax (Gibco) supplemented with 100 U/mL penicillin/streptavidin (Gibco) and 10% FCS (Sigma) per well. The 24-well plate with the biopsies was placed in an organ culture chamber (Billups Rothenberg) at 37°C with 95% O2 /5% CO2 atmosphere.

Biopsies were stimulated with 0.5 µM and 1 µM Allopurinol or the same concentrations of Febuxostat or left untreated (controls). Supernatants were collected and analyzed by ELISA assay; biopsies were used for IHC or protein extraction for Western blot analysis.

To exclude direct toxicity of the treatments, the viability of the biopsies over the culture period was checked by hematoxylin/eosin (H & E) staining of frozen tissue sections. The explants were considered viable only if the morphology of the tissue was intact with well-defined crypts, epithelial surface, and adequate and strong uptake of H & E.

*UHPLC-MS/MS analysis of uric acid*

The culture media were diluted using a solution of acetonitrile and water (95:5 v/v) containing 5% of formic acid. Subsequently, 50 μl from each sample was taken and solubilized in 150 μl of aqueous acetonitrile.

Chromatographic analysis of metabolites was done using a 1260 Agilent Infinity II multisampler BIO a binary pump. The chromatographic system was coupled to an Agilent Ultivo Triple Quadrupole mass spectrometer (Agilent Technologies, Santa Clara, USA). For the analysis we used an Acquity BEH Amide (1.7um 150 x 2.1 mm) column (Waters, Milford, MA, USA) at a constant temperature of 50°C while the mobile phase consisted of water and acetonitrile containing 0.1% of formic acid with a flow rate of 0.2 mL/min. The gradient was 99 % as initial condition, 0–1 min linear to 80%, kept for 7 min, and then linear to 30 % from 7 to 10 min, the system was equilibrated for 2 min at initial conditions.

Mass spectrometry analysis was performed using a Jetstream (ESI) operating in positive ion mode (except for uric acid). Nitrogen was used as sheath gas, drying gas, and collision gas. The sheath gas and drying gas flow rates were set at 11 L/min at 375 °C and 7 L/min at 300 °C, respectively. The nebulizer was set at 45 psi, and the capillary voltage and nozzle voltage were set at 4000 V and 1500 V, respectively. The fragmentor and cell acceleration voltage were set at 135 V and 9 V, respectively. The acquisition was performed in the MRM mode. The transitions used for the acquisition in MRM mode of the uric acid was 167 > 68.8m/z. The MS system was controlled by Agilent Mass Hunter Workstation Data Acquisition.

*Enzyme-linked immunosorbent assay (ELISA)*

In order to verify the release and concentration of cytokines, the culture media were carefully collected, centrifuged, and assayed with the following commercially available kit: Interleukin (IL1)beta (Invitrogen, Thermo Fisher Scientific # 88-7261-22), IL18 (MBL, Life Science #7620). Samples were tested according to the manufacturer's instructions, and their absorbance was measured at 450 nm using a microplate reader (Chameleon, Hidex).

*Western Blot*

Equal amounts of protein (20-40 µg) were mixed with Laemmli buffer, denatured at 95°C for 5 minutes, separated on a 10-12% SDS-PAGE gel, and transferred onto a PVDF membrane. The block of unspecific antibody binding was performed with 5% milk in TBS-T for 1 hour at room temperature. Membranes were incubated overnight at 4°C with primary antibodies (XDH 1:1000, NLRP3 1:1000, ASC – 1:500, Caspase-1 1:500, GAPDH 1:10000). After TBS-T washes, membranes were incubated with HRP-conjugated secondary antibodies (1:5000) for 1 hour and detected using ECL chemiluminescence. Bands were analyzed using the open source image processing software ImageJ and normalized against GAPDH band intensity.

*Immunohistochemistry*

Sigmoid colon and ileum tissue samples were fixed with 10% formalin- and paraffin-embedded. Serial microtome sections (5 μm thick) were treated for the immunohistochemical detection of XO, NPRL3, ASC1, and CASPASE-1 antigens, applying the streptavidin-biotin alkaline phosphatase method, as previously described (Murtas et al. 2019). Sections were dewaxed, rehydrated, and rinsed in phosphate-buffered saline (PBS), pH 7.4. Heat-induced epitope retrieval (HIER) was carried out by immersion in a water bath-heated 10 mM citrate buffer (pH 6.0) for 30 min at 95 °C, followed by gradual cooling for 20 min at room temperature (RT). Non-specific binding was inhibited by incubation in 10% non-immune serum for 45 min at RT in a humid chamber: normal goat serum (NGS; Sigma-Aldrich, St. Louis, MO, USA) for XO, NPRL3, and ASC1; normal horse serum (NHS; Sigma-Aldrich) for CASPASE-1. Then, the sections were incubated with the primary antibodies: rabbit monoclonal antibody to human XO (clone JG38-40, 1:200, 60 min at RT, MA5-34780, Thermo Fisher Scientific, Waltham, MA, USA); rabbit polyclonal antibody to human NPRL3 (1:300, overnight at 4 °C, PA5-98424, Thermo Fisher Scientific); rabbit polyclonal antibody to human ASC1 (1:200, 60 min at RT, PA5-90403, Thermo Fisher Scientific); mouse monoclonal antibody to human CASPASE-1 (clone 14F468, 1:200, 60 min at RT, MA5-16215, Thermo Fisher Scientific). Biotinylated goat anti-rabbit (1:200, 30 min at RT, BA-1000, Vector Laboratories, Burlingame, CA, USA) and horse anti-mouse (1:200, 30 min at RT, BA-2000, Vector Laboratories) antibodies were used as secondary antisera. Furthermore, the sections were treated with alkaline-phosphatase streptavidin (1:1000, Vector Laboratories) for 30 min at RT. Immunoreactivity was detected using the Fast Red substrate-chromogen system (Sigma-Aldrich), which develops the alkaline phosphatase reaction as a reddish product. All the sections were carefully rinsed in PBS after each step, finally counterstained by Carazzi’s haematoxylin, and mounted in glycerol gelatin (Sigma-Aldrich). Negative controls were carried out by omitting the primary antibodies to rule out the non-specific binding of secondary antibodies.

*Evaluation of immunohistochemical staining*

The immunostained slides were examined and semi-quantitatively scored for the distribution and intensity of staining by three observers (A.D.P., D.M., E.G.), independently, and the three assessments were averaged to provide the final value. For each sample analyzed, the percentage of immunoreactive cells and the degree of staining intensity were evaluated in five randomly chosen microscopic fields (x400 magnification), and the average of single counts was considered. Samples were then scored as negative (absence of immunoreactivity) or positive (presence of labelling with weak or moderate/strong intensity). Cellular compartments (nucleus, cytoplasm, or both) were analyzed for the localization of immunoreactivity.

Immunolabelled slides were analyzed using a Zeiss Axioplan2 microscope (Carl Zeiss Vision, Hallbergmoos, Germany), equipped with the 10×/0.25 Zeiss Achroplan, 20×/0.45 Zeiss Achroplan, 40×/0.75 Zeiss Plan-Neofluar, and 63×/1.40 oil immersion Zeiss Plan-Apochromat objectives. Image capture was performed by a Lumenera Infinity 3-1URC camera (1.4 megapixels; Lumenera Corporation, Ontario, Canada) and the Infinity Capture 6.3.0 software (Lumenera Corporation). Figures were slightly adjusted for brightness and contrast and arranged in panels by Adobe Photoshop CS3 Extended 10.0 software (Adobe Systems Incorporated, CA, USA).

*Caspase-1 activity assay*

Caspase-1 activity was assessed in the tissues lysates of organ cultures maintained for 24 hours either untreated or treated with xanthine oxidase inhibitors. The assay was performed using the Caspase-Glo® 1 Inflammasome Assay kit (Promega) according to the manufacturer’s instructions. Briefly, an equal volume of Caspase-Glo® 1 Reagent was added to each sample of culture supernatant, followed by gentle mixing and incubation at room temperature for 1 hour in the dark. Luminescence was then measured using a luminometer, and the signal intensity was proportional to the caspase-1 activity present in the samples. All luminescence was recorded on a GloMax® Multi+ Detection System.
