# Supplementary figures and images for: Xanthine Oxidase-Dependent Activation of NLPR3 Inflammasome in Epithelial Cells Sustains Inflammation in Inflammatory Bowel Disease
Source: Inflamm Bowel Dis. 2025 Oct 22;31(12):3398–406. doi: 10.1093/ibd/izaf231 (PMC12688067; doi:10.1093/ibd/izaf231)

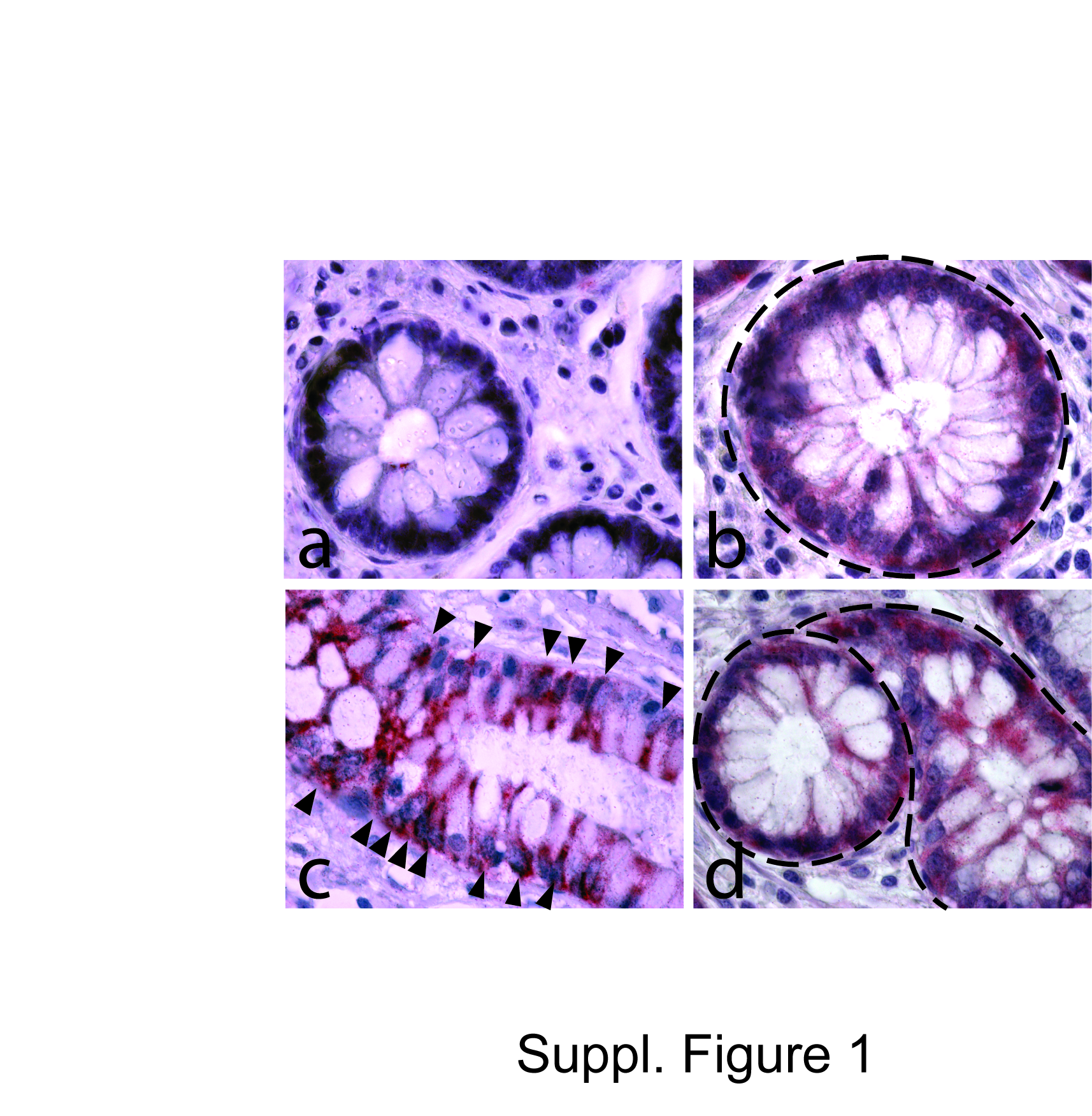

Supplement: izaf231_Supplementary_Data [file izaf231_supplementary_data.zip › Suppl.Fig1_2.0.tif]

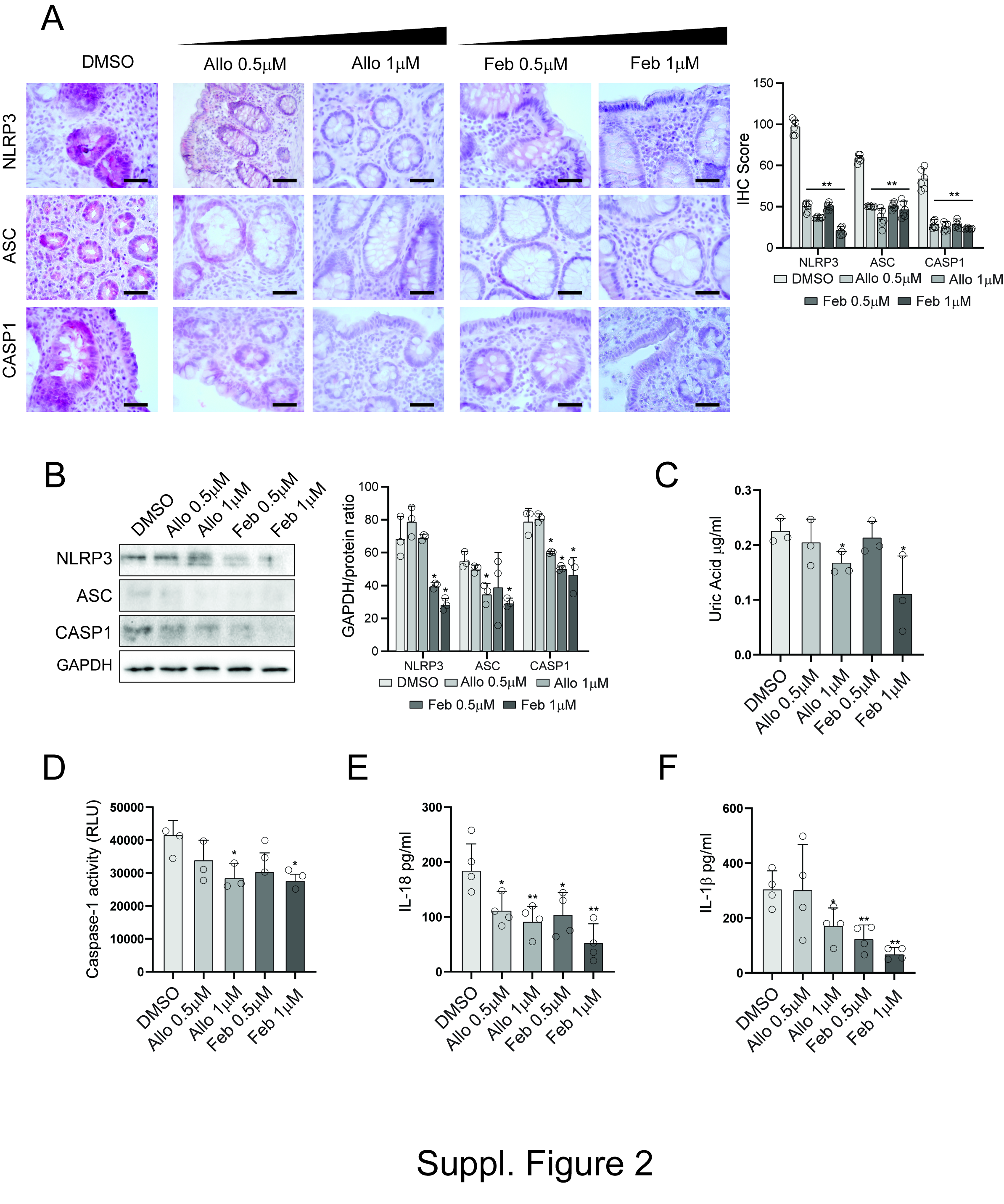

Supplement: izaf231_Supplementary_Data [file izaf231_supplementary_data.zip › Suppl.Fig2_2.0.tif]

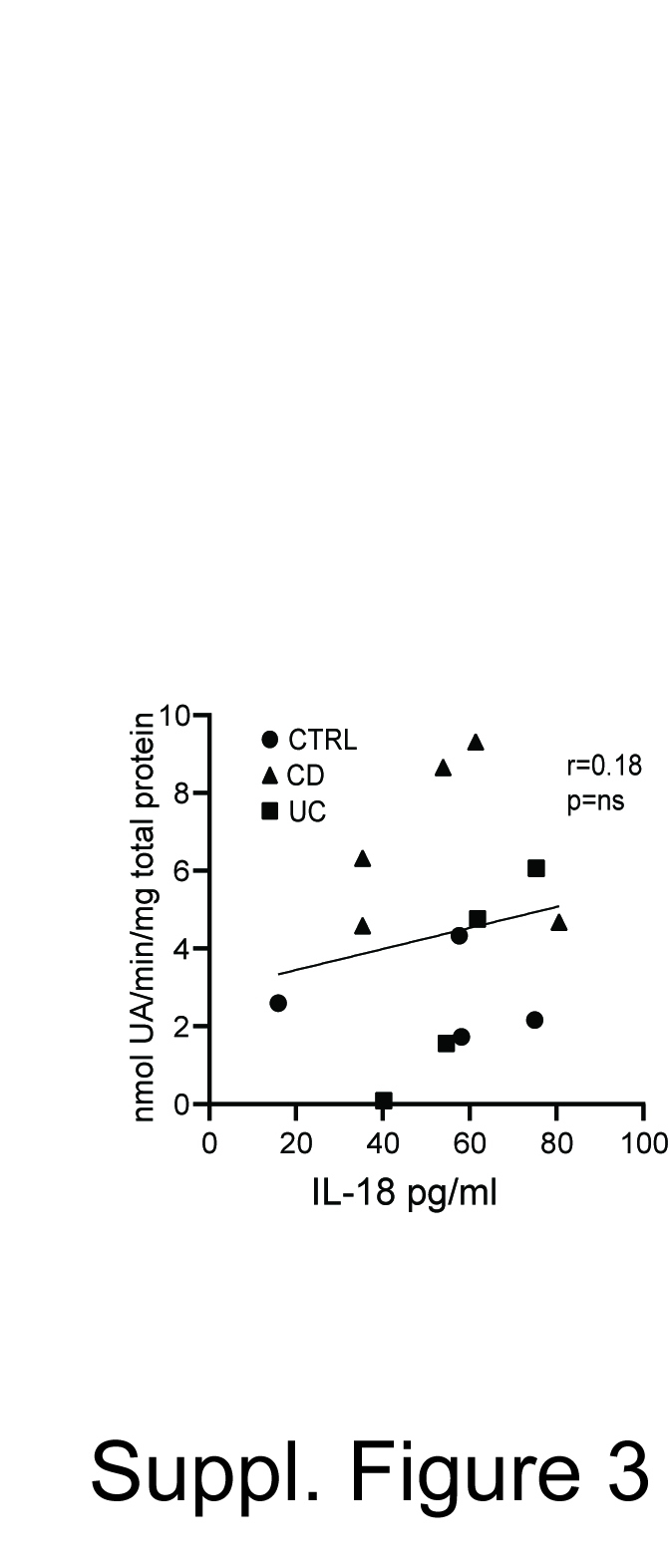

Supplement: izaf231_Supplementary_Data [file izaf231_supplementary_data.zip › Suppl.Fig3_2.0.tif]
